# Adherence to physical activity during the first trimester of pregnancy: a study from Southern Italy

**DOI:** 10.1007/s00404-026-08320-7

**Published:** 2026-01-18

**Authors:** Antonio Angelino, Dario Colacurci, Paola Borrelli, Anna Luna Tramontano, Ludovica Niccolini, Matteo Giudice, Mariella Calvanese, Cristina Mennitti, Olga Scudiero, Ilenia Mappa, Martina Derme, Giuseppe Rizzo, Maurizio Guida, Giuseppe Maria Maruotti, Laura Sarno

**Affiliations:** 1 Department of Public Health, University Federico II, Via Pansini 5, 80131 Naples, Italy; 2 Department of Medical, Oral and Biotechnological Sciences, Laboratory of Biostatistics, University G. D’Annunzio of Chieti-Pescara, Chieti, Italy; 3 Santa Maria Della Speranza Hospital, Battipaglia, Italy; 4 Department of Molecular Medicine and Medical Biotechnology, University Federico II, Naples, Italy; 5 Ceinge Biotecnologie Avanzate Franco Salvatore S. C. a R. L, Naples, Italy; 6 Task Force On Microbiome Studies, University Federico II, Naples, Italy; 7 Department of Maternal and Child Health and Urological Sciences, Sapienza University of Rome, Rome, Italy; 8 Department of Neurosciences, Reproductive Science and Dentistry, University Federico II, Naples, Italy

**Keywords:** First trimester, PPAQ, Physical activity, Pregnancy, Southern Italy

## Abstract

**Background:**

Although international recommendations strongly support regular physical activity during pregnancy due to the improved maternal and fetal outcomes, adherence to physical activity remains low, particularly in early gestation. Understanding activity patterns during the first trimester is crucial, as behaviors established in this phase often persist throughout pregnancy.

**Objectives:**

To describe physical activity levels and adherence to international recommendations among low-risk pregnant women in the first trimester, using the Italian version of the Pregnancy Physical Activity Questionnaire (PPAQ).

**Study Design:**

This cross-sectional observational study includes 498 low-risk singleton pregnant women between 11^+3^ and 13^+6^ weeks’ gestation, recruited at the University Hospital of Naples Federico II, Italy, between January 2022 and December 2023. Participants completed the Italian version of the PPAQ. Total energy expenditure was expressed in MET-h/week, and women were classified as *exercisers* (≥ 150 mines/week of moderate-intensity activity) or *non-exercisers*.

**Results:**

Participants reported a median of 11.5 (IQR 8.2–15.0) h/week of total activity, corresponding to 155.7 (102.6–241.7) METs-h/week. While 51% met the threshold of ≥ 150 min/week of moderate-intensity activity when considering all activity domains, only 7.8% reached this target through sport or structured exercise alone. Walking represented the most common exercise (64.1% slow, 46.2% brisk, 25.6% uphill). Employment status was significantly associated with higher adherence to recommendations, whereas other sociodemographic factors showed no significant differences.

**Conclusions:**

Structured exercise should be improved in the daily routine to optimize maternal and fetal health, although activity levels may appear adequate. Adherence to physical activity recommendations could be promoted by integrating validated tools such as the PPAQ into routine prenatal care and targeted interventions.

## What does this study add to the clinical work

**Table:** 

This study shows that during the first trimester most pregnant women meet physical activity recommendations mainly through daily activities rather than structured exercise. Routine use of validated tools such as the PPAQ during early prenatal visits can help clinicians identify insufficient exercise and deliver targeted counseling to promote safe, structured physical activity from early pregnancy.	

## Introduction

Regular physical activity before and during pregnancy is fundamental in promoting maternal and fetal health [1]. A growing body of evidence demonstrates that maintaining an active lifestyle is associated with a lower incidence of adverse outcomes such as gestational diabetes mellitus, hypertensive disorders of pregnancy, and excessive gestational weight gain [2]. Current international guidelines [1, 3, 4], including those issued by the World Health Organization [5] and the American College of Obstetricians and Gynecologists [6], recommend that pregnant women engage in at least 150 min per week of moderate-intensity aerobic exercise, complemented by resistance training and flexibility activities.

Despite these clear recommendations and the substantial evidence supporting the safety and benefits of physical activity during gestation [7], adherence remains disappointingly low [8], particularly in the early stages of pregnancy. According to the WHO Global Status Report on Physical Activity 2022 [9], more than a quarter of the global adult population fails to achieve the recommended levels of physical activity. In Italy, fewer than half of adults meet these targets [9] and among pregnant women the prevalence of inactivity is even higher. Many women tend to reduce or discontinue physical activity upon learning of their pregnancy, often due to concerns about fetal safety, lack of appropriate guidance, or symptoms such as fatigue, nausea, and anxiety [10].

While the literature has extensively examined exercise patterns during the later stages of pregnancy [11], data concerning physical activity in the first trimester remain limited. This gap is of relevance, as behaviors established in early pregnancy frequently persist throughout gestation. Moreover, the first trimester presents unique physiological and psychological challenges that may act as barriers to maintaining an active lifestyle [12]. Understanding these factors is therefore crucial for developing effective, evidence-based interventions aimed at promoting maternal well-being from the earliest phases of pregnancy.

To evaluate physical activity during pregnancy, several assessment tools have been proposed, with self-administered questionnaires representing the most practical and widely adopted approach in both research and clinical settings [13]. Among these, the Pregnancy Physical Activity Questionnaire (PPAQ) stands out as a validated and versatile instrument, available in multiple languages and adaptable to diverse cultural contexts [14]. Importantly, it captures a broad range of activities, encompassing not only recreational exercise, but also occupational, household, and caregiving tasks—domains that are particularly pertinent during pregnancy [2, 15].

The present study aims to describe adherence to physical activity among low-risk pregnant women in the first trimester, using the Italian version of the PPAQ [16], in a single-center population from Southern Italy. Through this analysis, we seek to provide a clearer understanding of exercise patterns and determinants of physical inactivity in early pregnancy, with the goal of informing targeted preventive and educational strategies to support maternal and fetal health.

## Materials and methods

### Study design and participants

This cross-sectional observational study was conducted between January 2022 and December 2023 at the Mother and Child Department of the University Hospital Federico II, in Naples, Southern Italy.

The study population consisted of singleton, low-risk pregnant women, recruited through a convenience sampling approach, during routine first-trimester ultrasound screening, between 11^+3^ and 13^+6^ weeks’ gestation.

A member of the research team invited women to participate while they were waiting for their scheduled consultation. After providing written informed consent, participants completed the Pregnancy Physical Activity Questionnaire (PPAQ) in a quiet, dedicated space.

Exclusion criteria included: multiple gestation, pre-existing chronic diseases (e.g., hypertension, diabetes mellitus, autoimmune conditions), current obstetric complications (e.g., threatened miscarriage, vaginal bleeding), and non-Italian speaking women or those unable to independently complete the questionnaire.

Several steps were taken to minimize potential sources of bias. Selection bias was limited by consecutively inviting all women during the first-trimester screening in the study protocol. To reduce information bias, all participants completed the PPAQ in a quiet, private environment, without external influence, and were encouraged to answer sincerely. The same trained member of the research team provided standardized instructions to ensure uniform administration of the questionnaire. To minimize transcription and processing errors, data entry and analysis were double-checked by two independent researchers. Finally, we excluded women with pre-existing chronic conditions to reduce confounding related to comorbidities.

This manuscript was prepared following the Strengthening the Reporting of Observational Studies in Epidemiology (STROBE) guidelines. The completed STROBE checklist is available as supplementary material.

### Physical activity assessment

An Italian version of PPAQ was used to assess physical activity levels.

The PPAQ is a semi-quantitative self-report instrument with 32 questions covering the following domains: 1. household/caregiving activities, 2. occupational activities, 3. transportation, 4. sport and exercise. A full version of the PPAQ has been previously published [17].

Participants reported the average time spent engaging in each activity during the current trimester, choosing from preset time intervals. The reported times were then converted into hours per week.

We assigned a metabolic equivalent of task (MET) value for each activity according to standardized compendia [18]. 1.0 MET is the energy expended in 1 min at rest, which is 3.5 ml of oxygen per kilogram of body weight per minute. Total weekly energy expenditure was calculated in MET-hours/week (METs-h/week) by multiplying the time spent on each activity by its corresponding MET value.

Activities were classified by intensity into: sedentary (< 1.5 METs); light (1.5 ≤ METs < 3.0); moderate (3.0 ≤ METs < 6.0); vigorous (≥ 6.0 METs).

Firstly, we reported the percentage of participants spending at least 150 min per week in moderate-intensity activities.

Secondly, according to adherence to physical activity recommendations (WHO, 2018; ACOG 2023),participants were therefore classified as “exercisers” if they reported at least 150 min per week of moderate-intensity sport/exercise activity, and as “non-exercisers” otherwise.

The PPAQ also captures sedentary behaviors (METs < 1.5), which were analyzed as a separate intensity category and were not included in the definition of moderate-intensity physical activity or in the classification of exercisers.

### Statistical analysis

Descriptive analysis was carried out using means and standard deviation (SD) or median and interquartile range (IQR) for the quantitative variables and absolute and percentage values for the qualitative ones.

The Shapiro–Wilk test was applied to assess normality. Univariate comparisons were investigated using the Pearson χ2 test or Fisher's exact test for categorical data and the non-parametric Wilcoxon rank-sum test for continuous data.

Statistical significance was taken at the < 0.05 level. All analyses were performed using Stata software v18.0 (StataCorp, College Station, Texas 77,845 USA).

Quantitative variables were summarized as means and standard deviations (SD) or medians and interquartile ranges (IQR), according to their distribution as assessed by the Shapiro–Wilk test. No multivariable analyses, subgroup evaluations, or sensitivity analyses were planned, as the study was primarily descriptive. There were no missing data in the main study variables. All eligible participants who consented to participate completed the questionnaire; therefore, no dropouts occurred. Values are reported as median (IQR). Domain- and intensity-specific times are not additive; therefore, their sum does not correspond to total weekly time.

### Ethics

The study was performed in accordance with the ethical guidelines of the Helsinki Declaration of the World Medical Association and was approved by the local ethics committee (University of Naples Federico II, IRB 483/21).

All the participants signed a written consent form before completing the questionnaire.

### Competing interest statement

The authors declare that they have no competing interests.

### Funding

The authors received no financial support for the research, authorship, and/or publication of this article.

## Results

### Study population

A total of 498 women with singleton low-risk pregnancies in the first trimester participated in the study.

The baseline characteristics of the study population are presented in Table 1.

**Table 1:** Baseline characteristics of the study population (BMI—body mass index)

	Total (*n* = 498)	Not exercisers (*n* = 459)	Exercisers (*n* = 39)	*p*-value
Age (years)	32.3 (5.5)	32.2 (5.5)	32.4 (5.3)	0.861
BMI (kg/m^2^)	24.8 (22.0–28.3)	24.8 (22.0–28.0)	25.7 (22.5–30.1)	0.114
BMI > = 30, n (%)				
< 30 kg/m^2^	401 (81.8%)	372 (82.5%)	29 (74.4%)	0.207
$\ge 30\text{ kg}/\mathrm{m}$ ^2^	89 (18.2%)	79 (17.5%)	10 (25.6%)	
Employed, n (%)				
Not employed	173 (35.2%)	168 (37.0%)	5 (13.2%)	**0.003**
Employed	319 (64.8%)	286 (63.0%)	33 (86.8%)	
Educational qualification, n (%)				
Primary school or lower	144 (29.6%)	137 (30.5%)	7 (18.4%)	0.283
Secondary school	181 (37.2%)	164 (36.5%)	17 (44.7%)	
Degree or higher	162 (33.3%)	148 (33.0%)	14 (36.8%)	
Total time	11.5 (8.2–15.0)	11.0 (8.0–14.8)	15.5 (11.8–21.5)	** < 0.001**
Total METs	155.7 (102.6–241.7)	153.1 (99.8–231.0)	241.6 (144.0–344.5)	** < 0.001**

Bold values indicate statistically significant data

^*^Data are expressed as mean (ds) or median (IQR) or *n* (%)

### Energy expenditure in the study population

According to PPAQ, our respondents spent 11.5 h/week (IQR 8.2–15.0) during the first trimester in all types of activities included in the questionnaire (household/caregiving, occupational, sport/exercise, transportation), corresponding to a total of 155.7 (IQR 102.6–241.7) METs-h/week.

Considering all the types of activities, 256 (51%) out of 498 participants reported spending at least 150 min per week in moderate-intensity activities.

However, only 39 participants (7.8%) achieved this target through structured sport/exercise activities alone.

For analytical purposes, participants were therefore classified as “exercisers” (*n* = 39) if they reported at least 150 min per week of moderate-intensity sport/exercise activity, and as “non-exercisers” (*n* = 459) otherwise.

Table 1 reports differences in baseline characteristics among exercisers and non-exercisers. Exercisers were significantly more often employed than non-exercisers. Groups did not differ for other characteristics.

Table 2 reports the time and METs spent by type and intensity. Both exercisers and not exercisers spent significantly more time and METs in household and caregiving activities.

**Table 2:** Metabolic equivalent of task (MET) spent by type and intensity

	Total (*n* = 498)	Not exercisers (*n* = 459)	Exercisers (*n* = 39)	*p*-value
By type of activities				
Household/caregiving time	8.5 (5.5–12.0)	8.5 (5.5–12.0)	8.5 (4.8–14.8)	0.861
Household/caregiving METs	128.8 (75.2–210.7)	128.3 (75.1–208.8)	140.5 (75.8–242.7)	0.440
Trasportation time	1.0 (0.5–1.8)	1.0 (0.5–1.8)	2.0 (1.8–3.0)	** < 0.001**
Trasportation METs	8.8 (5.2–17.5)	8.8 (5.2–17.5)	14.0 (5.2–31.5)	0.053
Occupational time	0.0 (0.0–0.0)	0.0 (0.0–0.0)	0.8 (0.0–6.0)	** < 0.001**
Occupational METs	0.0 (0.0–0.0)	0.0 (0.0–0.0)	15.8 (0.0–105.0)	** < 0.001**
Exercise time	0.0 (0.0–1.0)	0.0 (0.0–1.0)	3.0 (0.0–3.0)	** < 0.001**
Exercise METs	0.0 (0.0–23.0)	0.0 (0.0–21.0)	75.0 (0.0–94.0)	** < 0.001**
Sedentary time	3.0 (1.5–4.8)	3.0 (1.5–4.8)	2.2 (0.8–4.8)	0.152
Sedentary METs	23.7 (10.5–36.4)	24.1 (12.1–36.4)	15.8 (5.8–36.4)	0.119
By intensity				
Light time	5.2 (3.2–7.5)	5.2 (3.2–7.5)	6.8 (3.8–9.2)	**0.013**
Light METs	84.6 (48.8–125.7)	84.0 (47.8–124.4)	105.5 (62.6–155.1)	0.074
Moderate time	2.5 (0.8–4.8)	2.2 (0.8–4.2)	5.5 (4.5–9.8)	** < 0.001**
Moderate METs	47.2 (15.8–98.0)	42.0 (15.8–89.2)	101.5 (54.0–158.8)	** < 0.001**
Total time	11.5 (8.2–15.0)	11.0 (8.0–14.8)	15.5 (11.8–21.5)	** < 0.001**
Total METs	155.7 (102.6–241.7)	153.1 (99.8–231.0)	241.6 (144.0–344.5)	** < 0.001**

Bold values indicate statistically significant data

^*^Data are expressed as median (IQR), so domain- and intensity-specific times are not additive; therefore, their sum does not correspond to total weekly time

**Fig. 1:**
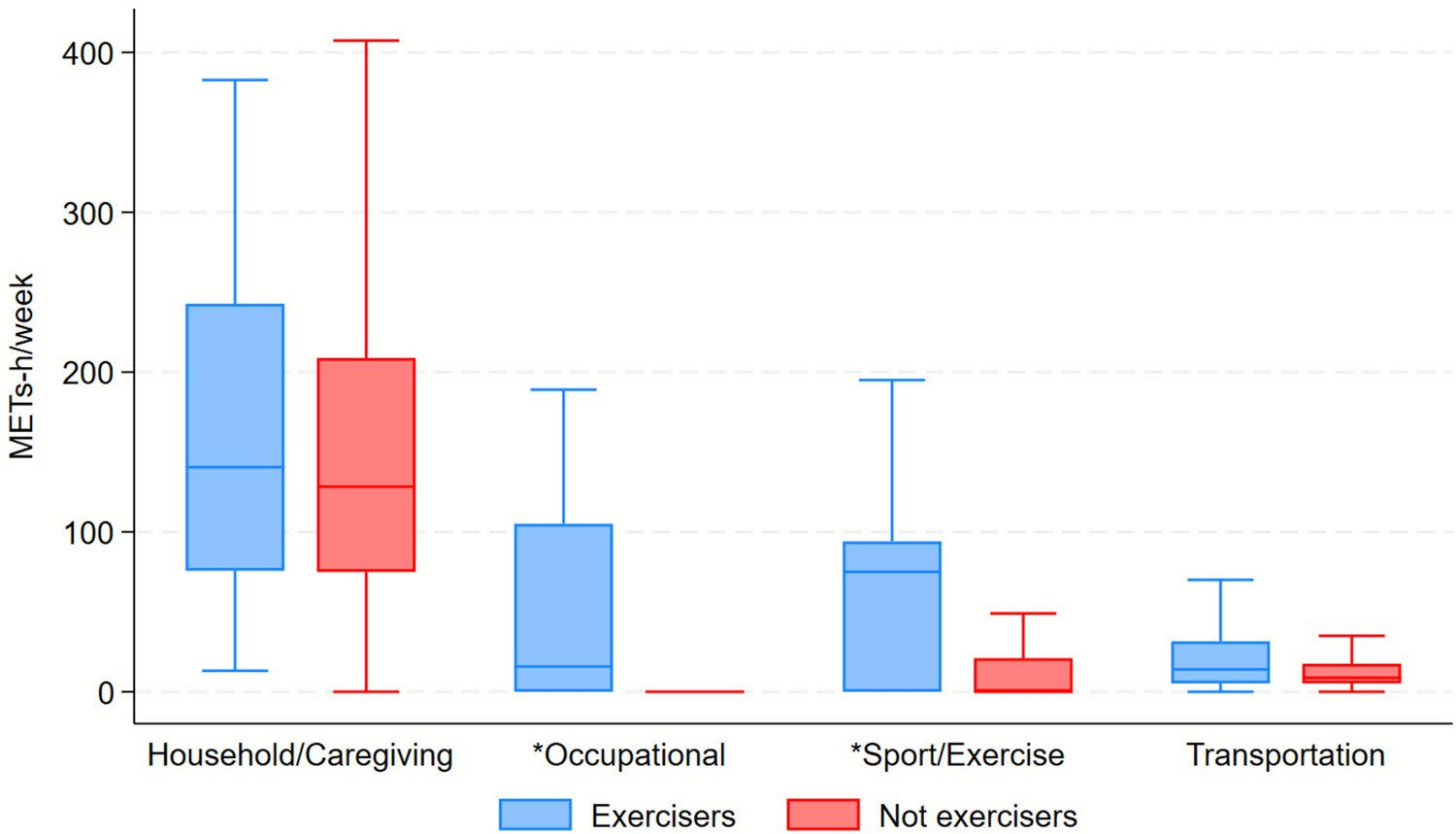
Distribution of energy expenditure (METs-h/week) according to activity category: not exercisers (n) = 459; exercisers (n) = 39; * indicates statistically significant differences between groups (*p* < 0.05)

**Fig. 2:**
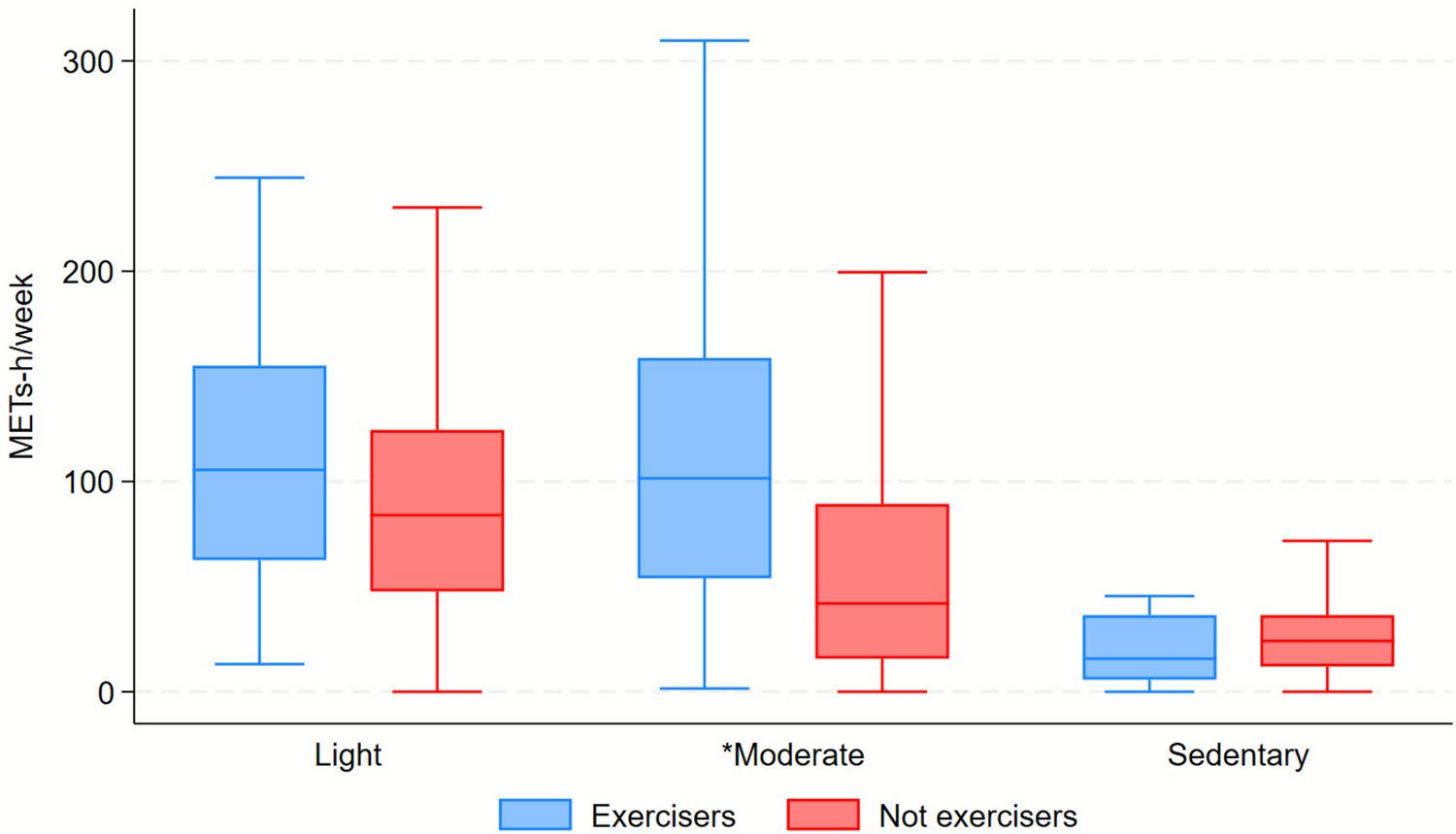
Distribution of energy expenditure (METs-h/week) according to different intensity of activities. Not exercisers (n) = 459; exercisers (n) = 39; * indicates statistically significant differences between groups (*p* < 0.05)

Figures 1 and 2 represent differences in energy expenditure according to type and intensity of activities.

Among exercisers, the main sporting activity during pregnancy was walking: 64.1% patients reported slow walking, 46.2% fast walking, and 25.6% uphill walking. Other activities included running (7.7%), yoga (5.1%), water aerobics (2.6%), and Pilates (2.6%).

To account for the risk of Type I errors arising from multiple comparisons, a Bonferroni adjustment was applied to the univariate analyses. For the baseline characteristics (Table 1), with seven comparisons, the adjusted significance threshold was set at alpha = 0.0071. Under this more stringent criterion, employment status (*p* = 0.003), total activity time (*p* < 0.001), and total METs-h/week (*p* < 0.001) remained statistically significant. For the detailed analysis of energy expenditure by type and intensity (Table 2), where 14 comparisons were performed, the adjusted threshold was set at alpha = 0.0035. The differences between exercisers and non-exercisers remained highly significant for transportation time (*p* < 0.001), occupational time and METs (*p* < 0.001), structured exercise time and METs (*p* < 0.001), and moderate-intensity time and METs (*p* < 0.001). Conversely, the difference in light-intensity activity time (*p* = 0.013) did not meet the adjusted threshold for significance.

## Discussion

In the present study, we describe a detailed overview of physical activity patterns among low-risk pregnant women in a single center in Southern Italy during the first trimester of pregnancy.

Despite the well-defined benefits of exercise during pregnancy, and despite the international guidelines [3, 4], our results indicate that compliance with these recommendations remains limited. Although the majority of the participants reported at least 150 min per week of moderate-intensity activity, fewer than one in ten achieved this target through exercise or sports alone. This underlines the predominant contribution of non-recreational activities, such as household chores and caregiving tasks.

These results align with previous studies: structured exercise is less commonly practiced while pregnant women often remain active in their daily routines [2]. Cultural and lifestyle factors play a key role in shaping the type and amount of physical activity undertaken during pregnancy. In Mediterranean contexts, this pattern may reflect both the persistence of traditional gender norms and the limited availability of accessible, pregnancy-specific exercise opportunities.

From a public health and clinical standpoint, the low prevalence of structured exercise observed is a cause of concern in our cohort. Regular, moderate-intensity physical activity before and during pregnancy is well established as beneficial for both maternal and fetal outcomes, contributing to a reduced risk of gestational diabetes mellitus, hypertensive disorders, excessive gestational weight gain, and cesarean delivery [7, 19]. In addition, physical activity supports psychological well-being by reducing symptoms of anxiety and depression and enhancing overall quality of life [20]. Despite this, our findings indicate that early pregnancy remains a critical “dropout” phase during which many women decrease or discontinue exercise. This could be explained by concerns about miscarriage, fatigue, or uncertainty regarding safe activity levels.

Nevertheless, the first trimester of pregnancy is a crucial period for behavioral intervention. Women’s habits develop during this time, often lasting through pregnancy and affecting long-term maternal and child health outcomes. In fact, early prenatal visits are a vital opportunity for healthcare providers to offer relevant counseling on physical activity. Additionally, our research found that employment status is linked to better adherence to physical activity recommendations. Employed women are more likely to be classified as exercisers than those who are not employed. This connection may reflect underlying socioeconomic and educational differences that impact lifestyle behaviors. Employed women might also benefit from more structured daily routines. However, it is important to recognize that occupational activity may not provide the same physiological protective benefits as recreational exercise, especially when it involves prolonged standing or repetitive tasks rather than sustained aerobic activity.

Walking is seen as the most common activity, practiced by more than half of those classified as exercisers. This result is coherent with global evidence indicating that walking is the preferred and most accessible form of physical activity during pregnancy [21]. It is a sustainable option for most women due to its low cost, flexibility, and safety. Nevertheless, the predominance of walking makes authors wonder if women are reaching sufficient exercise intensity to achieve optimal health status [22]. Other structured activities, such as prenatal swimming, yoga, and water aerobics, have demonstrated additional benefits. They support muscular strength, balance, and metabolic control and should be more actively promoted as complementary forms of physical activity [23–25].

Our research aimed to overcome a clear gap in the literature regarding physical activity during early pregnancy, especially in the first trimester. The majority of existing studies have focused on the second and third trimesters, where symptoms such as fatigue and nausea [19] typically subside. However, understanding women’s activity routines in the first trimester is essential. This period often influences future adherence and shapes lifestyle routines. Identifying barriers to physical activity at this stage could promote educational and behavioral interventions.

Our current findings during the first trimester seem to be coherent in certain aspects and contrasting in other aspects with those reported in our previous investigation conducted in the same center [17], which assessed physical activity in the third trimester using the same Italian version of the PPAQ. In that earlier study, only 37% of women achieved ≥ 150 min per week of moderate-intensity activity across all domains, with a very small proportion engaging in structured exercise. On the other hand, the present study showed a higher overall adherence (≥ 150 min/week in 51% of participants) and a slightly greater—but still insufficient—participation in structured exercise (7.8%). This discrepancy suggests that early-pregnancy engagement may be relatively better but tends to decline as gestation progresses, likely due to the combined influence of fatigue, medical advice, and lifestyle reorganization throughout pregnancy.

A second relevant difference concerns occupational status. In our previous study [17], third-trimester exercisers were more frequently homemakers, reflecting the dominant contribution of household and caregiving activities to overall energy expenditure. In contrast, in the current first-trimester cohort, employed women were more likely to meet activity recommendations. Although in the previous article we defined ‘exercisers’ as all patients who spent more than 150 min per week in moderate-intensity activities, despite the type of activity (occupation, transportation, household/caregiving, and sport). This could lead to a different evaluation of the comparison between the two groups.

This apparent inversion may have several non-mutually exclusive explanations: employed women who typically continue working in the first months of gestation may benefit from more structured daily routines and incidental activity; differences in recruitment context (screening visit versus late-pregnancy ambulatory access) could have influenced participation; and risk perception may shift with advancing gestation. Overall, when viewed together, these data highlight that behavioral determinants of physical activity are dynamic across trimesters and influenced by social and occupational context. Consistent with our previous findings, walking remains the most frequently reported activity among exercisers, confirming its accessibility and perceived safety. However, the persistently low proportion of women engaging in structured exercise across both studies underscores a continuing implementation gap: while women remain active through daily life tasks, few achieve the recommended duration and intensity of planned physical activity. This observation is particularly relevant given our earlier evidence of low counseling rates and the continued prescription of bed rest in our setting. Taken together, the comparison between the two studies suggests that early, structured counseling could help women maintain active behaviors throughout pregnancy, reducing the “dropout” in exercise adherence observed later in gestation. From a methodological perspective, the use of the same validated tool (PPAQ) in both studies represents a major strength, enhancing comparability. In our previous work, we also documented high test–retest reliability of the questionnaire, supporting its integration into routine antenatal care as a screening instrument. Systematic use of the PPAQ could help bridge the gap between incidental activity and structured exercise especially among non-employed women or those at behavioral risk of inactivity.

Differences in antenatal care pathways could partly explain the observed patterns of physical activity. Women consistently followed by the same team may have received more uniform, guideline-concordant messages on safe exercise, whereas those receiving care from multiple providers (inside and outside our hospital) might have been exposed to discordant advice (e.g., unnecessary exercise restrictions or bed rest suggestions). Although we did not measure continuity of care or the content of counseling, this potential heterogeneity may contribute to early “dropout” from structured exercise despite adequate activity in daily tasks. Embedding standardized, first-trimester counseling and reinforcing it across visits—irrespective of provider—could mitigate these inconsistencies.

The Pregnancy Physical Activity Questionnaire (PPAQ) appears to be an important and crucial system of evaluation in the first trimester, and it should be integrated into routine prenatal care. The PPAQ provides a validated, multidimensional evaluation of both recreational and non-recreational activities, facilitating clinicians’ ability to identify women at risk. Its use could help customize counseling and could allow the monitoring of longitudinal changes in activity levels throughout gestation. Furthermore, combining questionnaire-based assessments with objective measures, such as accelerometry [26], would increase accuracy and help validate self-reported data in future research. Likewise, combining the PPAQ with objective measures such as accelerometry could enhance accuracy and validation of self-reported data. Furthermore, multicenter and longitudinal studies are needed to assess changes in physical activity patterns across all trimesters and to generalize findings beyond a single Italian region.

### Strengths and limitations

The main strengths of this study include its relatively large sample size. Furthermore, while previous studies [17, 27, 28] reported Italian women’s attitudes toward physical activity later in pregnancy, to the best of our knowledge, this is the first study to provide data on the first trimester of pregnancy. Additionally, the use of a validated instrument specifically developed to assess pregnancy-related factors and the focus on an early gestational window aim to address a poorly studied aspect of the literature. By capturing multiple activity domains, our approach offers a more comprehensive understanding of women’s total energy expenditure, extending beyond just leisure activities and exercise during free time.

Nevertheless, several limitations should be acknowledged. The cross-sectional design limits causal inference and precludes the assessment of temporal changes in physical activity across trimesters. The reliance on self-reported measures introduces potential recall and social desirability bias. These could lead to either over- or underestimation of true activity levels. Furthermore, as recruitment was conducted at a single tertiary care center using a convenience sampling strategy, the findings may not be fully generalizable to all Italian or international populations, particularly those differing in socioeconomic status or access to prenatal services. Future multicenter, longitudinal studies are needed to validate and expand upon these findings.

The association between employment status and engagement in structured exercise was explored using univariate analyses only. We acknowledge that this association may be confounded by other unmeasured factors such as educational level, socioeconomic status, parity, BMI, pregnancy-related symptoms, and commuting patterns. The relatively small number of women classified as exercisers limited the feasibility of robust multivariable modeling. The relatively small number of women engaging in structured exercise also limited the statistical power of comparative analyses and precluded the detection of small effect sizes.

A further limitation of this study is the unequal size of the comparison groups, which may have limited the statistical power and reduced our ability to detect small but potentially meaningful differences between groups.

## Conclusions

In conclusion, this study shows that although a considerable proportion of low-risk pregnant women in Southern Italy maintain overall activity through domestic and caregiving tasks, participation in structured exercise during the first trimester remains markedly limited. Early pregnancy represents a critical, underutilized window for intervention. Healthcare professionals should proactively encourage safe, individualized physical activity from the earliest prenatal encounters. Counseling should emphasize the safety and benefits of exercise in uncomplicated pregnancies, offer practical examples of appropriate activities, and should address prevalent misconceptions and concerns.

## Data Availability

The datasets generated and/or analyzed during the current study are available from the corresponding author on reasonable request.
